# Drop Drying on the Sensor: One More Way for Comparative Analysis of Liquid Media

**DOI:** 10.3390/s20185266

**Published:** 2020-09-15

**Authors:** Tatiana Yakhno, Alexander Pakhomov, Anatoly Sanin, Vyacheslav Kazakov, Ruben Ginoyan, Vladimir Yakhno

**Affiliations:** 1 Institute of Applied Physics RAS, 46 Ulyanov Street, 603950 Nizhny Novgorod, Russia; b9050116845@rambler.ru (A.P.); agsanin@mail.ru (A.S.); kazak@appl.sci-nnov.ru (V.K.); yakhno@appl.sci-nnov.ru (V.Y.); 2 Nizhny Novgorod State Agricultural Academy, 97 Gagarin Ave, 603107 Nizhny Novgorod, Russia; r.ginojan@yandex.ru; 3 N. I. Lobachevsky State University of Nizhny Novgorod (National Research University), Institute of Information Technologies, Mathematics and Mechanics, 23 Gagarin Ave, 603950 Nizhny Novgorod, Russia

**Keywords:** drying drops, sensor device, signal processing, comparative analysis, recognition statistics

## Abstract

It is known that the processes of self-organization of the components of drying a liquid drop on a solid substrate are well reproduced under the same external conditions and are determined only by the composition and dispersion of the liquid. If the drop dries on the surface of the sensor device, these processes can be recorded and used as a passport characteristic of the liquid. The first half of the article is devoted to the description of the principles of the method and the proof of the validity of our assumptions. The second half of the article is devoted to the development of a user-friendly version of the device, where the change in the real and imaginary parts of the electrical impedance of the resonator was used as an informative parameter. The measure of the closeness of the relative positions of the hodographs of the compared samples on the complex plane is used as a criterion for the similarity-/-difference of various liquids. The design of a new sensor device and the results of its tests for distinguishing between different brands of alcoholic beverages and reconstituted milk of different concentrations are presented.

## 1. Introduction

### 1.1. The Morphological Characteristics of Dried Drops as a Source of Information about the Liquid Quality

In the middle of the last century, Leonard Bolen, a medical doctor from the Hall River General Hospital (MA, USA) proposed a test for the rapid diagnosis of oncological diseases [1,2]. The test was made as follows. A patient’s finger was pricked with a needle, and a glass slide was applied onto the drop of the released blood. A thin layer of blood—an imprint—remained on the glass. The dried imprint was examined in transmitted light with naked eye or with a small magnification. A sign of the presence of a malignant disease, regardless of the tumor location, was a cellular structure of the imprint containing empty spaces—lacunas. No lacunas were observed in healthy individuals and patients without oncology, and their imprints had a homogeneous fine-grained structure. According to the results of the diagnostic studies conducted by the author, the information content of the test was close to 100%. It is interesting that such a cellular structure of blood imprints was observed in women in late pregnancy and returned to norm after childbirth. Obviously, neoplasm appearance and growth in the body was accompanied by characteristic changes in the physical properties of the blood, which was manifested in the nature of structuring of the drying thin layer. Further independent trials gave contradictory results and the colleagues did not approve this test for use in medical practice. It should be mentioned that the necessary condition of the test was that the layer of blood on the glass should not be too thick. Perhaps not all colleagues adhered to these recommendations.

The interest in droplets of biological fluids dried on glass as an object of additional medical diagnosis increased in Russia after the publication of the book by Shabalin and Shatokhina *“Morphology of Biological Fluids”* [3], where it was shown that, under room conditions, the pattern of the droplets dried on glass was reproducible and based on the chemical composition of biological fluids, which could be used as an additional diagnostic criterion. The only factor that reduced the express advantages of the method was the need for a long (at least two days) preliminary drying of the drops under room conditions to obtain a clear image. The strict dependence of the structure of droplets dried on glass on the composition, concentration of components, and quality of the substrate was proved experimentally and theoretically by different researchers [4,5,6,7,8,9,10,11,12]. The methods for the recognition, registration, and quantitative processing of structural patterns in dried droplets that are characteristic of liquids of different compositions were proposed [13,14,15]. Kim et al. [15] developed a computer-based classification and clustering of dried drops images using pattern-recognition algorithms. Using this technique, the authors recognized images of drying drops via an inverted microscope as signatures of fluid composition for rapid biological testing. Two data sets of drying drops images were prepared, one with consumable fluids (beers, juices, liquors, milk, red wines, and carbonated drinks) and the other with different buffer solutions. One can hardly regard this analysis as quick and inexpensive, as the drops should dry for at least 24 h before analysis, and the analysis is performed using an inverted microscope and special cameras. The need for expensive equipment and long pretreatment of samples reduces their research capabilities and consumer advantages of this method.

### 1.2. The Dynamic Characteristics of Drying Drops as a Source of Information about the Liquid Quality

Thus, it was shown that the analysis of structures in dried droplets of liquids, regardless of their nature, is informative with respect to the chemical composition of these liquids. However, the analysis was long and required expensive equipment. We decided to investigate whether it is possible to obtain diagnostic information faster—during the drying process of the drops. Below are the results of these studies, confirming the correctness of our assumption. Historically, we started our research with human biological fluids.

At the beginning of this century, we set the task to find out whether the dynamic parameters of drying drops of blood serum have diagnostic information content. The observations were carried out under a microscope, registering every minute of the progress of the droplet front structuring from the periphery to the center up to complete evaporation of free water [16]. The path of the structuring front and the speed of its advancement were determined. The recorded diagnostic indicators proved to be quite informative. However, the procedure for obtaining them was very laborious. There arose the idea to verify whether it is possible to obtain diagnostic information using acoustic impedancemetry during drying a drop on the surface of a quartz resonator. Our technological approach relies on the following logical chain. A drop of liquid drying on a solid wettable substrate is a natural model of a self-organizing system with an infinitely wide variety of dynamics of molecular self-assembly processes depending on the composition and structure of the liquid. The initial physicochemical parameters of the solution affect the dynamics of the processes such as coacervation, precipitation, sedimentation, gelation, and crystallization that accompany the drying process of a multicomponent liquid. As a result, the physical properties of the drop change and their dynamics can be registered. Under the same external conditions (including the substrate), these dynamics are determined only by the composition and structure of the liquid. We constructed a laboratory prototype of the device and convincing evidence of the legitimacy of such an idea was obtained [17]. A detailed description of the prototype design, signal registration, and parametrization of the results were presented in [18,19].

## 2. Materials and Methods

### 2.1. Fundamentals of the Method Used

The device allows recording the dynamics of the mechanical properties of the droplets drying on the surface of a quartz resonator using acoustic impedance measurements. The recorded value is called “acoustomechanical impedance” (AMI) [18]. AMI measurements are based on the dependence of the electrical characteristics of a resonator on the physical properties of a liquid. This dependence is widely used in the studies of the properties of gases and liquids by means of electro-acoustic resonators QCM [19,20,21,22]. The measured electrical characteristics of a resonator are, as a rule, its resonance frequency and Q-factor, which change when the resonator contacts the object under study. This property is usually used as the basis for determining the physical properties of a liquid such as viscosity, density, concentration of sought substance, and so on. A radical difference of our method from the known ones is that we use the temporal dependence of the AMI of the drying drop as the informative parameter [18]. At the same time, the resonator oscillation frequency of 60 kHz is kept constant during measurements. Part of the area of the electrodes at the operating end of the plate was removed to place the drop directly on the surface of a quartz crystal. In that case, the drop is an acoustic (mechanical) load of the resonator for shear oscillations. The amplitude of shear oscillations of quartz does not exceed 10 nm. Under such conditions, there are no significant violations of the structuring of the studied drops in comparison with the control, which was confirmed experimentally [20]. A detailed description of the prototype design, signal registration, and parametrization of the results were presented in [18,19]. Here we provide the basic information needed for understanding the gist of the technology and some results that have not been published before.

### 2.2. Amplitude Curve Parametrization (Shape Indices) as a Tool for Distinguishing between Liquids

**Figure 1:**
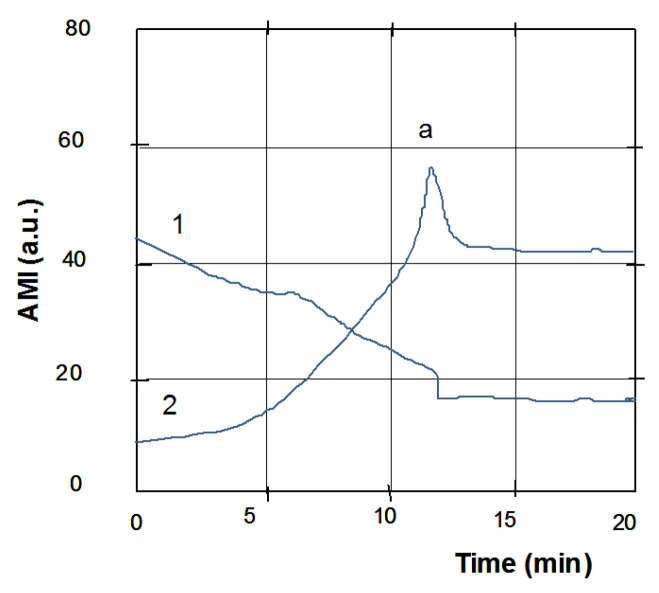
Change in the amplitude (1) and phase (2) of acoustomechanical impedance (AMI) during drying of a drop of tested fluids on the sensor surface.

Drying of the droplet is accompanied by a regular change in its physical characteristics, which is reflected in the corresponding changes in the electrical parameters of the quartz resonator. The module and phase of the electrical impedance of quartz or the real and imaginary parts of the quartz impedance can be the recorded. All these parameters change in time as the droplet is drying. The recording of any of them is, in fact, an oscillogram of the test liquid drying. During the droplet drying and transition to the solid state, the mechanical interaction of the droplet residues with the sensor increases and manifests itself in an increase in the signal of the module and a decrease in the signal of the AMI phase. Upon reaching the ultimate mechanical stress (critical point, a), the signal decreases as a result of microcracks in the dried material and its partial delamination [21] (Figure 1). This is the basic pattern of the change in the module and phase of AMI for all aqueous solutions. However, changes in the geometry and amplitude of the signals depend on the composition, concentration, and dispersion of each liquid, which allows the differences between the liquids to be assessed quantitatively.

**Figure 2:**
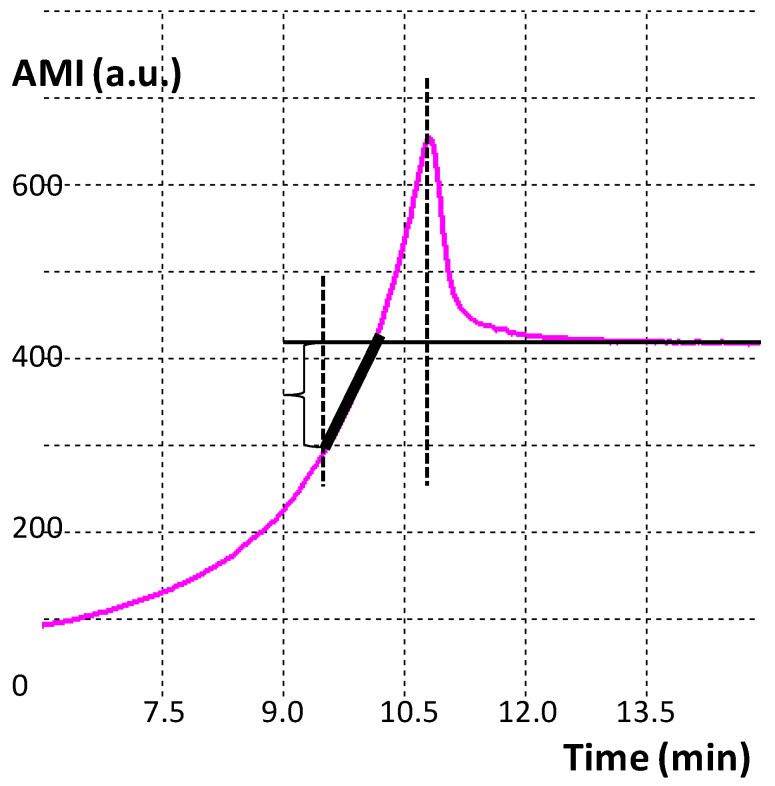
Calculation of SI-1: finding reference points and determining design parameters characterizing the shape of the amplitude curve of a drying drop. SI-1 is the total derivative on the section of the curve marked in black.

An indispensable stage of processing the curves is choosing their informative areas. At this stage of work, we analyzed the shape of the amplitude component of the AMI in the area of its peak (Figure 1a) [19]. Eighteen heuristic algorithms were developed—the amplitude curve shape indices (SI), which made it possible to parameterize the obtained data and compare them quantitatively on the plane of features in the coordinates of certain algorithms. When calculating shape indices, parameters such as the characteristic time scale of the curve in the peak area, the derivatives of the rising and falling branches, the area under the peak, etc., were used (Figure 2). Various ratios of these parameters made it possible to obtain several heuristic algorithms with the help of which the distinction was carried out (see Supplementary Materials).

This approach was laid in the software of the first version of our device (prototype). With its help, results were obtained confirming the viability of our technology. By replacing proteins and salts in protein–salt solutions substantially, we found the contribution of each component to the dynamics of AMI and the structural features of the droplets dried on glass [20,21,22,23,24,25]. With the use of the technology encouraging results were obtained in differential diagnosis of diseases [17], in assessing the degree of endogenous intoxication in patients with burn disease [26], in veterinary diagnostics [27], in wine recognition, including the blind method [28,29], detecting fake drugs [25], and registering of odors and effects on solutions of external factors of physical nature [30,31].

## 3. Next Generation Device

### 3.1. Technical Features of the Next Generation Device

**Figure 3:**
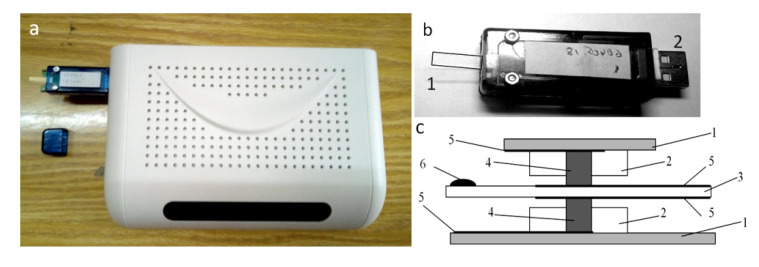
(**a**) Photo of a modified version of the device, overall dimensions: 200 × 140 × 50 mm, weight 200 g. (**b**) Sensor module detached from the device body: 1—quartz resonator and 2—USB-connector. (**c**) Diagram of the device of the sensor module: 1—printed circuit board, 2—plastic parts, 3—quartz plate with metallization, 4—conductive rubber, 5—metallization layer, and 6—drop.

The device was constructed from modern components, which made it possible to significantly reduce its size and make it more compact (Figure 3a). The sensor module is made detachable by attaching it to the device body using the USB port (Figure 3b). The sensor mount was strengthened by the use of additional parts (Figure 3c).

In the prototype design, the sensor was suspended on thin wires—electrodes [18], which created difficulties in cleaning it from the remnants of dried droplets and often led to breakage of contacts. In the new version of the device, the sensor was mounted in its central part using conductive rubber and was made detachable for ease of processing. This inevitably led to a loss in the quality factor of the sensor of ~25%, which required additional tests to assess the sensitivity of the sensor to distinguish liquids of different compositions.

### 3.2. Signal Registration

**Figure 4:**
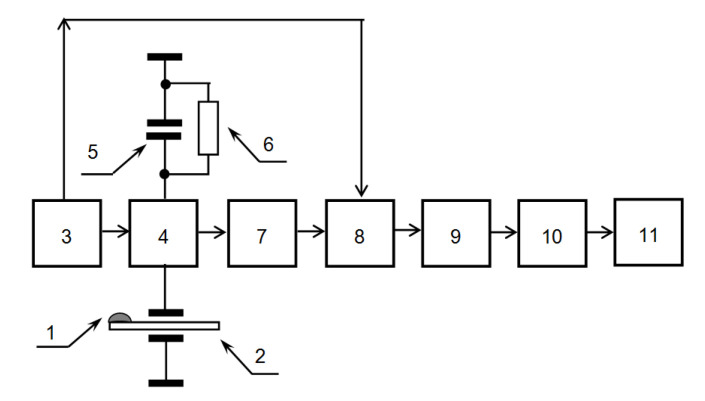
Functional diagram of the device: 1—drop; 2—sensor; 3—generator; 4—bridge circuit; 5—compensating capacitor; 6—reference resistor; 7—unbalance voltage amplifier; 8—phase detectors; 9—integrators; 10—analog-to-digital converter; and 11—computer.

Simultaneous registration of two parameters, namely, the real Re (Z) and imaginary Im (Z) parts of the electric impedance Z of the sensor allows the representation of the results in the form of a hodograph—a curve on the plane in the coordinates of the real and imaginary parts of the electric impedance. Such a representation of changes of the electrical impedance of quartz during drop drying in the form of oscillogram is also a “fingerprint” of the liquid. A new interface was developed: both the real and imaginary parts of electric impedance of the sensor are used for analysis. The electronic unit of the device has a built-in microcontroller and is designed to convert the signal from the quartz resonator to voltages corresponding to the real and imaginary parts of its electrical impedance, which change during the drying process (Figure 4). The output voltage can be converted into the mechanical characteristics of a drying drop; however, for a comparative analysis of liquids, such a conversion is not needed. The data is transferred via USB to a laptop, where it is visualized, processed, and stored.

### 3.3. Signal Processing

**Figure 5:**
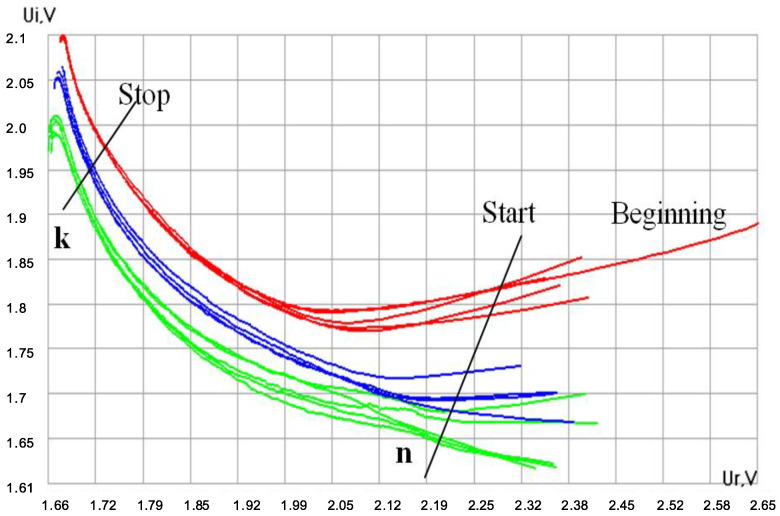
An example of calculating the difference index (DI) of two hodographs as the average distance between the points of the corresponding samples: n is the starting point of reference; k is the end point of reference.

The viewer of stored data files permits displaying the stored data and make a preliminary comparison of the “fingerprints” of different liquids visually, with the “fingerprints” of one type of liquid being displayed in one color. The software for comparing the catalogs of dynamic portraits calculates the distances between the hodographs related to a series of repeated measurements of the drying process of one liquid and between the hodographs related to two different liquids. This allows comparing the catalogs of “fingerprints” of two liquids inside the database to reveal statistical differences between them. The hodograph difference index (DI) is calculated as the average distance between the points of the corresponding samples of two hodographs (Figure 5).

**Figure 6:**
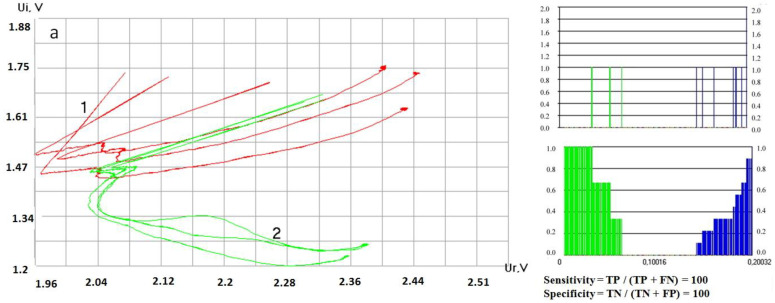
Software interface. (**a**) Hodographs of electrical impedance: sample 1—port wine “777” (Russia) and sample 2—vermouth “Martini Bianko” (Italy). (**b**) Intermediate data for calculating proximity measures between sample 1 and sample 2: green shows the distance between hodographs for sample 1; blue shows distances between hodographs for sample 1 and hodographs for sample 2. Ordinate - axis is the number of measurements for given DI values. The abscissa is the DI values. (**c**) Joint distribution of the estimates for errors of the second kind (green) and errors of the first kind (blue) versus DI value with the calculation of sensitivity and specificity [32].

The DI of the hodographs is calculated by Equation (1).

(1) $$DI=\frac{\sum_{i=n}^{k}\sqrt{{{(x}_{i1}{-x}_{i2})}^{2}+{{(y}_{i1}{-y}_{i2})}^{2}}}{k-n}$$

where x_il_ is the x coordinate of the first hodograph, x_i2_ is the x coordinate of the second hodograph, y_i1_ is the y coordinate of the first hodograph, y_i2_ is the y coordinate of the second hodograph, *n* is the starting point of calculation, and *k* is the end point of calculation. When two identical datasets were compared, the DI was zero. The more differences between the hodographs, the larger the DI magnitude is. Using the automatically constructed hodographs, it is possible to estimate quantitatively the extent of the difference between the liquids of these two groups taking into account errors of the first and second kind (level of intersection of the corresponding histograms, Figure 6).

Despite the noticeable spread between repeated measurements associated with the non-stationary processes of drying of liquids with two solvents—water and alcohol—the shapes of the curves of the same liquid are self-similar, and the difference between the samples is obvious. On completion of the calculations, a list of liquids in the group sorted in ascending order of the averaged minimum distances and the “Result” window in which the liquid with the greatest similarity to the tested one is indicated can be displayed on the monitor (not shown). This enables finding liquids in the group the hodographs which are located closest to the tested one, and therefore having the greatest similarity with it.

Thus, a new version of the device based on the drying drop technology was developed, more robust, user-friendly and providing clear statistical characteristics of fluid comparison—sensitivity and specificity. It required a test of technology carried out by “alien hands” in another territory. Students of higher educational institutions of Nizhny Novgorod took part in the tests of the new device.

### 3.4. Results of Validation

Whole milk powder, wine, and hard drinks were bought in the city shops (Table 1). The experiments were carried out in laboratories at room temperature (20–23 °C) and humidity (40–60%). Before each entry, current file name was set in the working window of the program. The registration software automatically tuned the frequency in the ±1 Hz range for approximately 40 s. Upon completion of the automatic frequency tuning, a drop of the studied liquid with a volume of 3 μL was placed using a micropipette on the surface of the sensor, which served as a signal to start recording. Recording ended at the end of the time set by the operator. For each test liquid, 3–7 drops were measured. After each measurement, the remnants of the dried drop were carefully washed off the surface of the quartz plate with a cloth moistened with distilled water, wiped with a dry cloth and then with an alcohol wipe. After that, the next measurement was made. Twenty-five samples were used in the study, including hard drinks, red and white wine, and milk (Table 1).

**Figure 7:**
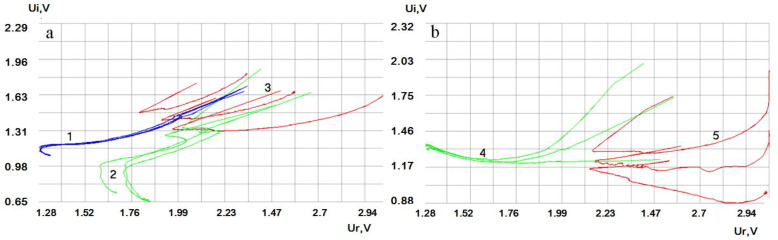
Software interface (**a**,**b**). A variety of shapes of hodographs of liquids of different classes: 1—dry red wine; 2—cognac; 3—whiskey; 4—balm; and 5—vodka.

The results of the study depicted in the form of hodographs of electrical impedance of quartz on a complex plane demonstrate a wide variety of shapes depending on the type of drink (Figure 7).

The recognition statistics, Sensitivity and Specificity, based on the calculation of the hodographs DI of the compared samples and errors of the first and second kind are presented in Table 2.

According to the results of the tests (Table 2), the new implementation of the method demonstrated sensitivity sufficient for distinguishing drinks and a number of user advantages over the previous version. An unexpected result was low sensitivity of distinguishing between Georgian brandy Eristavi 3* and Russian brandy Staraya Krepost, which indicates the significant similarities of these drinks.

## 4. Discussion

In this paper, we presented a new approach for rapid assessment of the similarity/difference of compared drinks—multicomponent liquids—without determining their composition. The information content of our method is based on the high sensitivity of the electrical characteristics of the oscillating sensor to the dynamics of the physical properties of a drop of the test liquid drying on its surface. As a result, each liquid acquires its own “dynamic portrait” (imprint), which reflects the component composition and dispersion of the liquid under given (room) conditions.

**Figure 8:**
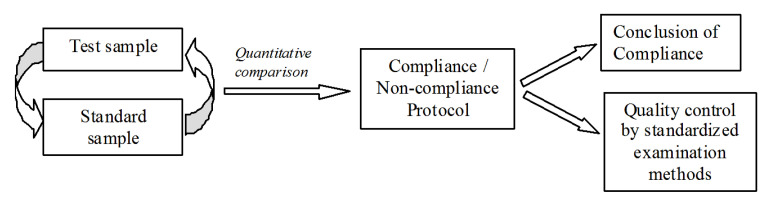
Scheme of incorporating the proposed method into the procedure of assessing the compliance of a liquid with its standard.

As far as we understand, nature has worked under the problem of a food recognition system for several thousand years. The best instrument for solving it is the human natural sensor system that combines nose, tongue, and brain. Working together, they are capable of immediately creating from thousands of separated feelings one unique image, and remembering it. The only drawback of this method is that this image cannot be digitized [28]. In the drying drop technology that we have been developing in the past decade, we used the same holistic approach. It uses only one integral parameter, which is its ID characteristic. This is a dynamic parameter arising from the processes during drying of a droplet of tested liquid on the quartz sensor surface. This parameter can be expressed in numerical form, which allows quantitative comparison of different samples. No additional reagents and sample pretreatment are required. A droplet, a sensor surface, and software are all one needs. Thus, our approach combines a sensor technology and self-organization processes in sessile drying drops. In different implementations of the method, the fingerprint may be represented either by the shape of the amplitude curve (SI) or the difference index (DI) in the relative position of the hodographs on the complex plane, indicating the statistical differences between them—Sensitivity and Specificity. In either case, the extent of the difference between the liquids is expressed quantitatively. The scientific novelty of our approach was confirmed by the patents [33,34,35]. Depending on the user need, the proposed device can be used to analyze any liquids (compare them with the corresponding standards). The only limitation is that liquids must evaporate under room conditions. We believe that the considered quick and accurate evaluation of the similarity or difference of compared liquids is highly promising for preliminary rapid assessment of the quality of liquids, in particular, juices and wines, which can speed up and reduce the volume and cost of the quality assessment procedure using methods of standardized examination (Figure 8). This will require creating specialized databases of reference samples collected in advance by the interested user. The coverage of possible falsifications with the use of this method will be much wider than that ensured by the control of individual indicators proposed by a number of researchers for specific samples [36,37]. If there is a sufficient number of databases of reference liquids, the closeness of a certain sample to one or another database will mean that it belongs to a particular class of liquids. This is a real way to liquid certification. The dynamic portrait (fingerprint) of a liquid could replace the barcode on product packaging, since the label on the packaging, strictly speaking, does not correlate with the quality of the liquid inside it and can also be falsified.

The advantages of the method also include absence of preliminary treatment of samples, comparison of results at room temperatures and moderate humidity, compactness and transportability of the device, its low cost, versatility, ease of use, and environmental safety.

## 5. Conclusions

We have presented a new approach to the comparative analysis of liquids and the result of the tests on the example of distinguishing alcohol products. A dynamic indicator—an oscillogram of the change in the electrical impedance of the sensor on which a drop of the studied liquid dries—is used as a “fingerprint” of a multicomponent liquid. We have described the design and principle of operation of the developed device, as well as examples of its testing. The proposed approach is efficient for distinguishing samples not only between different types of wine products, but even within each type, such as cognacs of different varieties, dry and semi-dry red and white wines of different brands, and milk of different concentrations. The method is simple, does not require preliminary sample preparation, and works in the range of room temperature and humidity fluctuations. The device has small dimensions and weight and can be used as a compact console for a laptop. The authors believe that the dynamic “fingerprint” can replace the widely used barcode and form the basis for certification of wine products.

## Figures and Tables

**Table 1:** List of the samples under study.

	Hard Drinks		Wine
1.	Georgian brandy Eristavi 5*	1.	Semi-sweet red table wine Toro de Oro (Spain)
2.	Georgian brandy Eristavi 3*	2.	Port wine 777 (Russia)
3.	Russian brandy Staraya Krepost	3.	Vermouth Martini Bianco (Italy)
4.	Russian brandy Lezginka	4.	Semi-sweet grape natural red wine Khvanchkara (Georgia)
5.	Russian drink Three old men	5.	Dry white table wine Chardonnay (Fanagoria, Russia)
6.	Irish whiskey Jameson	6.	Dry white table wine Sauvignon (Fanagoria, Russia)
7.	Macallan Whiskey (Scotland)	7.	Dry white table wine Aligote-Riesling of Fanagoria (Fanagoria, Russia)
8.	Mordovian balm (Russia)	8.	Semi-dry red wine Pirosmani (Georgia)
9.	Brandy Stareishina (Russia)	9.	Semi-dry red wine Pirosmani (Georgia) + sugar (3 g/100 mL)
10.	Vodka Khlebnaya Sleza (Russia)	10.	Semi-dry red wine Pirosmani (Georgia) + sugar (6 g/100 mL)
		11.	Semi-dry red wine Pirosmani (Georgia) + sugar (9 g/100 mL)
Milk			
1.	Whole powdered milk, 6 g/100 mL distilled water	3.	Whole powdered milk, 10 g/100 mL distilled water
2.	Whole powdered milk, 8 g/100 mL distilled water	4.	Whole powdered milk, 14 g/100 mL distilled water

**Table 2:** The obtained differences between the compared samples.

Compared Samples		Recognition Statistics (EER—Equal Error Rate)	
Hard drinks		Sensitivity	Specificity
1.	Georgian brandy Eristavi 5* and Georgian brandy Eristavi 3*	93	99
2.	Georgian brandy Eristavi 5* and Russian brandy Staraya Krepost	97	100
3.	Georgian brandy Eristavi 3* and Russian brandy Staraya Krepost	20	99
4.	Russian brandy Lezginka and Russian drink Three old men	100	100
5.	Russian brandy Lezginka and Irish whiskey Jameson	100	100
6.	Macallan Whiskey (Scotland) and Mordovian balm (Russia)	100	100
7.	Vodka Khlebnaya Sleza (Russia) and Brandy Stareishina (Russia)	100	100
Wine			
8.	Semi-sweet red table wine Toro de Oro (Spain) and Port wine 777 (Russia)	100	100
9.	Port wine 777 (Russia) and Vermouth Martini Bianco (Italy)	100	100
10.	Semi-sweet red table wine Toro de Oro (Spain) and Semi-sweet grape natural red wine Khvanchkara (Georgia)	100	100
11.	Dry white table wine Chardonnay (Fanagoria, Russia) and Dry white table wine Sauvignon (Fanagoria, Russia)	100	100
12.	Dry white table wine Sauvignon (Fanagoria, Russia) and Dry white table wine Aligote -Riesling of Fanagoria (Russia)	100	100
13.	Semi-dry red wine Pirosmani (Georgia) and Semi-dry red wine Pirosmani (Georgia) + sugar (3 g/100 mL)	100	100
14.	Semi-dry red wine Pirosmani (Georgia) + sugar (3 g/100 mL) and Semi-dry red wine Pirosmani (Georgia) + sugar (6 g/100 mL)	100	100
15.	Semi-dry red wine Pirosmani (Georgia) + sugar (6 g/100 mL) and Semi-dry red wine Pirosmani (Georgia) + sugar (9 g/100 mL)	100	100
Milk			
16.	Whole powdered milk, 6 g/100 mL distilled water and Whole powdered milk, 8 g/100 mL distilled water	99	85
17.	Whole powdered milk, 6 g/100 mL distilled water and Whole powdered milk, 10 g/100 mL distilled water	100	100
18.	Whole powdered milk, 10 g/100 mL distilled water and Whole powdered milk, 14 g/100 mL distilled water	100	100

## Supplementary Materials

All the data can be found in data repository: Hard Drinks (https://github.com/TVK-dev/TVK-data/tree/master/hard_drincs), Wine (https://github.com/TVK-dev/TVK-data/tree/master/wine), Milk (https://github.com/TVK-dev/TVK-data/tree/master/Milk_database), Shape Indices (https://github.com/TVK-dev/TVK-data/tree/master/Appendix).
